# PAID CAREGIVERS PROVIDE SOCIAL SUPPORT TO FAMILY CAREGIVERS OF OLDER ADULTS WITH DEMENTIA

**DOI:** 10.1093/geroni/igad104.1201

**Published:** 2023-12-21

**Authors:** Emma Quach, Emily Franzosa, Lauren Moo, Christine Hartmann

**Affiliations:** VA Bedford, Bedford, Massachusetts, United States; Icahn School of Medicine at Mount Sinai, New York City, New York, United States; Veterans Health Administration, Bedford, Massachusetts, United States; VA Bedford Healthcare System, Bedford, Massachusetts, United States

## Abstract

Family caregivers support older adults with dementia to age in place but often experience caregiver strain, though sharing the care with paid caregivers could reduce such strain. We investigated the potential synergy between family caregivers and paid caregivers. In summer 2021, we conducted 14 interviews with family caregivers (6), home care workers (3), and social workers who work with family caregivers and home care agencies (5). We audio-recorded, transcribed, and thematically analyzed our interviews. Our analysis revealed 4 types of support family caregivers received from paid caregivers: 2 centering on the family caregiver as an individual (“person-centered”) and 2 on the relational role the individual holds as a family caregiver (“relationship-centered”). (1) Family caregivers felt positive, emotional bonding with paid caregivers, describing them as “part of the family”. (2) Family caregivers valued instrumental aid from paid caregivers, paradoxically for tasks that are beyond their formal responsibilities, e.g., feeding pets. (3) Family caregivers recognized and valued expert personal care provided to their loved ones by highly experienced paid caregivers. (4) Family caregivers valued paid caregivers’ sharing information about their loved ones and their living situations, especially when family caregivers lived separately from their loved ones. Paid caregivers may reduce or prevent family caregiver strain by providing person- and relationship-centered to family caregivers. Home care agencies should foster the relationship between paid caregivers and family caregivers.

